# The Effects of Housing Density on Social Interactions and Their Correlations with Serotonin in Rodents and Primates

**DOI:** 10.1038/s41598-018-21353-6

**Published:** 2018-02-22

**Authors:** Young-A Lee, Tsukasa Obora, Laura Bondonny, Amelie Toniolo, Johanna Mivielle, Yoshie Yamaguchi, Akemi Kato, Masatoshi Takita, Yukiori Goto

**Affiliations:** 1 Department of Food Science and Nutrition, Daegu Catholic University, Gyeongsan, Gyeongbuk 38430 South Korea; 2 Primate Research Institute, Kyoto University, Inuyama, Aichi 484-8506 Japan; 3 École Nationale Vétérinaire de Toulouse, Toulouse, 31076 France; 4 Brain Function Measurement Research Group, National Institute of Advanced Industrial Science and Technology, Tsukuba, Ibaraki 305-8566 Japan; 5 Brain Science Inspired Life Support Research Center, The University of Electro-Communications, Chofu, Tokyo 182-8585 Japan

## Abstract

Population density has been suggested to affect social interactions of individuals, but the underlying neural mechanisms remain unclear. In contrast, neurotransmission of monoamines such as serotonin (5-HT) and dopamine (DA) has been demonstrated to play important roles in social behaviors. Here, we investigated whether housing density affected social interactions of rodents and non-human primates housed in groups, and its correlations with monoamines. Japanese macaques exhibited higher plasma 5-HT, but not DA, concentrations than rhesus macaques. Similarly, C57BL/6 mice exhibited higher plasma and brain tissue 5-HT concentrations than DBA2 mice. Under crowding, C57BL/6 mice and Japanese macaques exhibited more prominent social avoidance with mates than DBA2 mice and rhesus macaques, respectively. Although DBA2 mice and rhesus macaques in crowding exhibited elevated plasma stress hormones, such stress hormone elevations associated with crowding were absent in C57BL/6 mice and Japanese macaques. Administration of parachlorophenylalanine, which inhibits 5-HT synthesis, increased social interactions and stress hormones in C57BL/6 mice under crowding. These results suggest that, animals with hyperserotonemia may exhibit social avoidance as an adaptive behavioral strategy to mitigate stress associated with crowding environments, which may also be relevant to psychiatric disorder such as autism spectrum disorder.

## Introduction

Population density can be a potent environmental factor that affects brain functions through various mechanisms. Studies have shown that not only social isolation^1^, but also crowding^2^ causes stress, which in turn affects cognitive and affective functions^3^, in subjects living in such social environments. On the other hand, “cognitive overloads” has also been proposed to affect behaviors of subjects living in crowding environments^4^. Thus, exceeding amounts of sensory stimuli and processing associated with crowding could affect their behaviors in the way to reduce such sensory processing demands, for instance, by decreasing social interactions with others in crowding social groups. Consistent with this scheme, several primate species such as chimpanzees^5^ and capuchins^6^ have been shown to exhibit lower social interactions without alterations of distress levels when they are housed in higher housing density than those in lower housing density conditions, whereas other primate species such as rhesus macaques do not exhibit decreased social interactions, but elevations of stress, with increasing housing density^7^.

Neurotransmission of monoamines such as dopamine (DA) and serotonin (5-HT) in the mesocorticolimbic pathways has been demonstrated to entail social behaviors and social cognition of animals and humans. For instance, amphetamine administration has been shown to suppress social interactions in rodents^8,9^ and non-human primates^10–15^, through over-stimulation of D1 receptor^12,13^. In contrast, amphetamine could also facilitate social interactions in humans^16,17^, although the dose of amphetamine administration in human studies was even lower than those used in animal studies. Although acute administration of serotonin reuptake inhibitor (SSRI), which increases 5-HT transmission, has been shown to attenuate social interactions in normal rodents^18,19^, it also paradoxically facilitates social interactions in a rodent model of psychiatric disorder that exhibits impaired social behaviors^20^. In human studies, both decrease and increase of 5-HT transmission by tryptophan depletion^21^ and SSRI treatments^22^, respectively, have been demonstrated to facilitate social affiliations. DA and 5-HT transmission could also inversely be modulated by social environments. For instance, both social isolation^1^ and crowding^23,24^ have been shown to induce stress responses in rodents, which in turn affect DA and 5-HT transmission. Thus, DA and 5-HT mediates social behaviors, and social environments in turn modulate social behaviors through modulation of DA and 5-HT transmission, constructing reciprocal relationships between social behaviors and social environments^25^.

Collectively, housing density may affect social behaviors of individuals living in social groups through alterations of monoamine transmission. In this study, we investigated the effects of housing density on social interactions and the monoamine systems in rodents and non-human primates with different genetic backgrounds. We first examined social interactions of group housed Japanese and rhesus macaques, and then in rodents to evaluate whether the findings in rodents and primates were consistent. In rodents, two different strains, C57BL/6 (C57) and DBA2 (DBA), of mice, were examined, as C57 mice are more sensitive to sensory stimuli (higher sensory startle response and weaker sensory gating) than DBA mice^26^. Thus, if cognitive overloads are involved in alterations of social interactions in crowding, C57 mice were expected to show more prominent decrease of social interactions than DBA mice. C57 mice and Japanese macaques, whose plasma 5-HT concentrations and coritcolimbic 5-HT tissue concentrations (5-HT availability) were higher than DBA and rhesus macaques, respectively, exhibited more active social avoidance under crowding than dispersing conditions, whereas such alterations were less clear in DBA mice and rhesus macaques. Moreover, although DBA mice and rhesus macaques exhibited increased stress under crowding, such stress associated with crowding was absent in C57 mice and Japanese macaques.

## Results

### Social interactions and monoamine concentrations in primates

**Figure 1:**
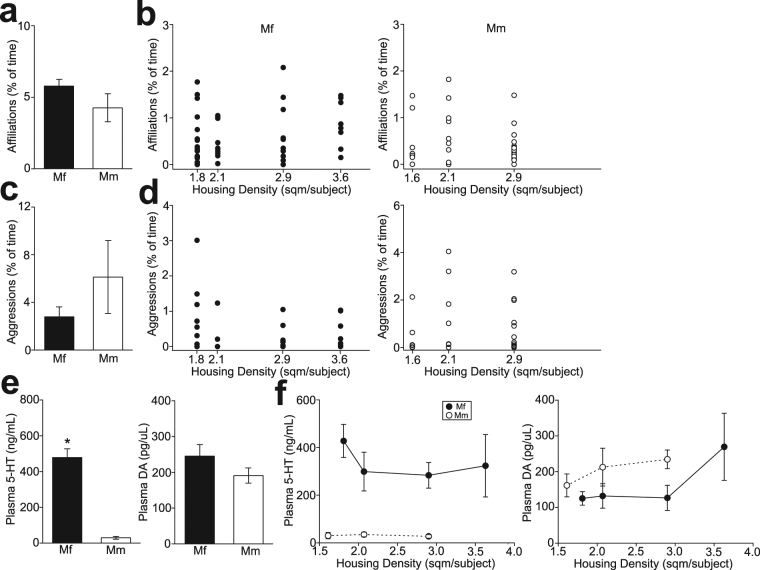
Social interactions and plasma monoamine concentrations in group-housed macaques. (**a**) A graph showing overall percentages of time that Japanese (Mf) and rhesus (Mm) macaques spent social affiliations. Error bars indicate mean ± s.e.m. (**b**) A graph showing the relationship between housing density and percentages of time that Japanese (left) and rhesus (right) macaques spent social affiliations on each observation day. (**c**,**d**) Graphs similar to (**a**) and (**b**), but showing aggressions. (**e**) A graph showing plasma 5-HT (left) and DA (right) concentrations in Japanese and rhesus macaques. *p < 0.001 for Mf and Mm. (**f**) Graphs showing the relationships between housing density and plasma 5-HT (left) and DA (right) concentrations in group-housed Japanese and rhesus macaques.

Behavioral observations were conducted in 5 Japanese (*Macaca fuscata*; Mf; n = 31 in total) and 4 rhesus (*Macaca mulatta*; Mm; n = 31 in total) macaque social groups with different housing density (from 1.6 to 3.6 m^2^/subject). There was no difference in overall numbers of social affiliations (unpaired t-test, t_7_ = 1.541, p = 0.167; Fig. 1a) and aggressions (t_7_ = 1.185, p = 0.275; Fig. 1c) between Japanese and rhesus macaque social groups. However, a significant monotonic decrease in the number of social affiliations was observed with increasing housing density in Japanese (n = 50, 5 groups × 10 observation days per cage; Spearman’s rank-order correlation, R = 0.309, p = 0.029; Fig. 1b), but not in rhesus (n = 40, 4 groups × 10 observation days per cage; R = 0.077, p = 0.638; Fig. 1b) macaque groups. No correlation between aggressions and housing density was found in both Japanese (R = 0.212, p = 0.140; Fig. 1d) and rhesus (r = −0.132, p = 0.896; Fig. 1d) macaque groups.

Plasma 5-HT and DA concentrations in Japanese (n = 54 including those not subjected for behavioral observations) and rhesus (n = 31) macaques were examined using enzyme-linked immunosorbent assay (ELISA) and high-performance liquid chromatography (HPLC), respectively. Two groups in each of Japanese and rhesus macaques, respectively, were the same housing density, and plasma monoamine and stress hormone concentrations of subjects between these 2 groups with the same housing density in each species were combined, since no statistically significant difference was observed in these chemical assay data (Suppl. Table S1). Plasma 5-HT concentrations were substantially higher in Japanese than rhesus macaques (t_85_ = 7.111, p < 0.001; Fig. 1e), whereas plasma DA concentrations were not different between them (t_85_ = 1.237, p = 0.220; Fig. 1e). Plasma 5-HT and DA concentrations were also not different across different housing density within the same species (Fig. 1f).

These results suggest that Japanese macaques, which have higher plasma 5-HT concentrations than rhesus macaques, exhibit decreased social interactions under higher housing density.

### Social interactions in rodents

Although a causal relationship was unclear from the above observations in primates, if 5-HT is associated with decreased social alterations under high social density environments, similar observations are also expected in other species of animals. Thus, we investigated the effects of housing density on social interactions of C57 (C57BL) and DBA (DBA/2) mice that were grouped into 2, 4, or 8 mice per cage (denoted as 2mpc, 4 mp, and 8 mpc, hereafter) by observations of pairs of mice in their home cages.

**Figure 2:**
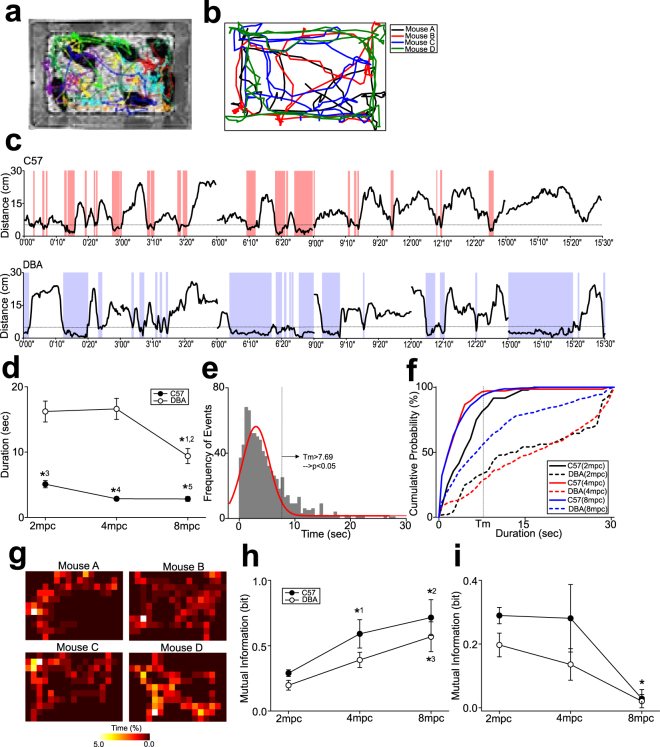
The effects of housing density on social interactions of mice in their home cages. (**a**) A photograph illustrating an example of tracking of mice under the housing condition of 8 mice per cage (8 mpc). (**b**) A diagram illustrating an example of tracking of mice under 4 mpc without backgrounds. (**c**) Diagrams illustrating examples of distance changes between each pair of C57 and DBA mice over time, with 6 times of 30 second recordings in 15 minutes. Red and blue screens indicate the periods in which distance between a pair of mice reached the threshold (i.e. less than 5 cm). (**d**) A graph showing duration of social interactions in C57 and DBA mice under different housing density. *^1^p = 0.034 for 2 mpc vs. 8 mpc in DBA, *^2^p = 0.046 for 4 mpc vs. 8 mpc in DBA, *^3^p < 0.001 for C57 vs. DBA under 2 mpc, *^4^p < 0.001 for C57 vs. DBA under 4 mpc, *^5^p = 0.024 for C57 vs. DBA under 8 mpc. (**e**) A graph showing a normal distribution of duration for the distance reaching less than 5 cm by chance, which was calculated by randomly shuffled pairs of mice. *Tm* indicates the duration at 2 standard deviations from the mean of the normal distribution, corresponding to statistical significance (p < 0.05). The red line indicates Gaussian curve fitting to the distribution. (**f**) A cumulative histogram for duration of social interactions shown in (**d**), along with *Tm* shown in (**e**). (**g**) Diagrams illustrating an example of a group of 4 mice for probability of locations that each mouse was observed within the home cage during observations. (**h**) A graph showing the amount of mutual information in each pair of mice. *^1^p = 0.002 for 2 mpc vs. 4 mpc in C57, *^2^p < 0.001 for 2 mpc vs. 8 mpc in C57, *^3^p = 0.006 for 2 mpc vs. 8 mpc in DBA. (**i**) A graph similar to (**h**), but showing the amount of mutual information in whole groups. *p = 0.040 for 2 mpc vs. 8 mpc in C57.

Mice in home cages were tracked using the tracking software (Fig. 2a,b). Social interactions of any kinds (including both social affiliations and aggressions) were defined as distance of a pair of mice reaching less than 5 cm. Durations of such instances at every 30 seconds for 6 times of observations was calculated (Fig. 2c). Group averages of social interactions in C57 mice were found substantially lower than those in DBA mice (n = 48, 8 cages under 2 mpc x 6 observations; n = 16, 4 cages under 4 mpc x 6 observations; n = 12, 2 cages under 8 mpc × 6 observations, in each of C57 and DBA mice; two-way ANOVA, F_1,162_ = 67.6, p < 0.001 for strain; F_2,162_ = 3.95, p = 0.021 for housing density; F_2,162_ = 2.06, p = 0.131 for interaction; post-hoc Tukey test, p < 0.001 for C57 vs. DBA under 2 mpc and 4 mpc, p = 0.024 for C57 vs. DBA under 8 mpc; Fig. 2d). DBA mice exhibited significant decrease of social interactions with increasing housing density (p = 0.034 for 2 mpc vs. 8 mpc, p = 0.046 for 4 mpc vs. 8 mpc; Fig. 2d), whereas C57 did not, most likely due to the floor effect (Fig. 2d). Since it is possible that distance of a pair of mice reaches less than 5 cm by chance, we further calculated such extrinsic duration by analyzing randomly shuffled pairs of mice. This analysis demonstrated that if social interactions happened at approximately 7.7 seconds or longer in a 30 second epoch, such interactions could be beyond the chance level at p < 0.05 (Fig. 2e). Such higher than the chance level of social interactions were 66.7%, 71.5%, and 44.6% of the cases in DBA mice under 2, 4, and 8 mpc, respectively (Fig. 2f). In contrast, in C57 mice, only 18.7%, 3.5%, and 6.5% of the cases were higher than the chance level under 2 mpc, 4 mpc, and 8 mpc, respectively (Fig. 2f).

We further investigated whether social interactions between a pair of mice did not happen at mere chance, but with any intentions. This was achieved with Shanon’s information theory to calculate mutual information that each pair of mice had (Fig. 2g–i). Although duration of social interactions decreased as housing density increased, group averages of the amount of information in pairs of mice were oppositely increased both in C57 and DBA mice, with higher information in C57 than DBA mice (F_1,162_ = 6.07, p = 0.015 for strain; F_2,162_ = 19.3, p < 0.001 for housing density; F_2,162_ = 0.499, p = 0.608 for interaction; Fig. 2h). Post-hoc analysis revealed that the amount of information in pairs of mice under 4 mpc (p = 0.002) and 8 mpc (p < 0.001) were significantly higher than that under 2 mpc in C57 mice (Fig. 2h). The amount of information under 8 mpc was also higher than that under 2 mpc in DBA mice (p = 0.006; Fig. 2h). In contrast, when the amount of information was calculated for entire groups, decreased information was found as housing density increased (F_1,160_ = 2.71, p = 0.102 for strain; F_2,160_ = 6.06, p = 0.002 for housing density; F_2,160_ = 0.495, p = 0.611 for interaction; Fig. 2i). In particular, the amount of information under 8 mpc was significantly lower than that under 2 mpc in C57 mice (p = 0.040).

These results suggest that social interactions of mice are decreased as housing density is increased. Such effects appears to be more prominent in C57 than DBA mice.

### Sociability test in rodents

**Figure 3:**
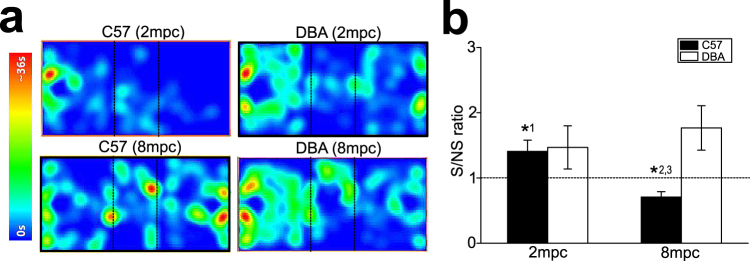
The effects of housing density on sociability of mice in three-chamber test. (**a**) Examples of heat maps showing time and locations in the chamber that C57 and DBA mice under 2 mpc and 8 mpc conditions spent in the three-chamber test. (**b**) A graph showing the ratio of duration to stay at social (S) over nonsocial (NS) sides of the camber. *^1^p = 0.0499 relative to S/NS ratio of 1.0, *^2^p = 0.010 relative to S/NS ratio of 1.0, *^3^p = 0.029 for 2 mpc vs. 8 mpc in C57.

Alterations of social interactions by crowding in C57 and DBA mice were further evaluated using three-chamber sociability test (Fig. 3a). The ratio of time spent in social and nonsocial sides of the chamber (S/NS ratio) was compared between C57 and DBA mice under 2 mpc and 8 mpc (n = 8 for each strain and housing density). In this test, significant difference was found between C57 and DBA mice (F_1,28_ = 4.93, p = 0.035 for strain) but not in housing density (two-way ANOVA, F_1,28_ = 0.557, p = 0.462 for housing density). Marginally significant difference was found for their interaction (F_1,28_ = 3.92, p = 0.058 for interaction; Fig. 3b). Post-hoc analysis revealed significant difference in the S/NS ratio of C57 mice between 2 mpc and 8 mpc (p = 0.029; Fig. 3b). Significantly longer stay in the social over nonsocial sides was observed in C57 mice under 2 mpc (one-sample t-test for above or below the ratio of 1.0, t_7_ = 2.37, p = 0.0499; Fig. 3b). In contrast, significantly shorter stay in the social over nonsocial sides was observed in C57 mice under 8 mpc (t_7_ = −3.51, p = 0.010; Fig. 3b). In DBA mice, although not statistically significant, a trend of longer stay in the social than the nonsocial sides was observed under 8 mpc (t_7_ = 2.29, p = 0.056; Fig. 3b), but not under 2 mpc (t_7_ = 1.42, p = 0.199; Fig. 3b).

These results suggest that housing density affects sociability of C57 mice, but much less extent in DBA mice.

### Monoamine availability in rodents

**Figure 4:**
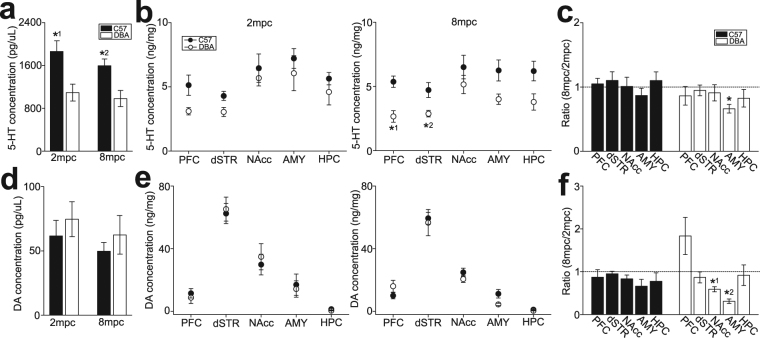
Plasma monoamine concentrations and monoamine availability in corticolimbic regions of group-housed mice. (**a**) A graph showing plasma 5-HT concentrations in C57 and DBA mice under 2 mpc and 8 mpc. *^1^p = 0.008 for C57 vs. DBA under 2 mpc, *^2^p = 0.044 for C57 vs. DBA under 8 mpc. (**b**) A graph showing tissue 5-HT concentrations (5-HT availability) in mesocorticolimbic regions of C57 and DBA mice under 2 mpc and 8 mpc. *^1^p = 0.006 for C57 vs. DBA in PFC, *^2^p = 0.016 for C57 vs. DBA in dSTR. (**c**) A graph showing the ratio of 5-HT availability in each brain region for 8 mpc over 2 mpc conditions. *p = 0.001. (**d**) A graph similar to (**a**), but showing plasma DA concentrations. (**e**,**f**) Graphs similar to (**b**) and (**c**), but showing DA availability. *^1,2^p < 0.001.

We examined plasma 5-HT and DA concentrations of C57 and DBA mice under 2 mpc and 8 mpc, using HPLC. Consistent with non-human primates, plasma 5-HT concentrations in C57 mice were significantly higher than those in DBA mice, regardless of housing density (two-way ANOVA, F_1,28_ = 19.8, p < 0.001 for strain; F_1,28_ = 1.47, p = 0.235 for housing density; F_1,28_ = 0.257, p = 0.616 for interaction; p = 0.008 for C57 vs. DBA under 2 mpc, p = 0.044 for C57 vs. DBA under 8 mpc; Fig. 4a), whereas plasma DA concentrations were not different between C57 and DBA mice in both housing conditions (Fig. 4d).

To identify monoamine transmission of which brain regions may be involved in alterations of social interactions by housing density, we investigated tissue concentrations of DA and 5-HT (DA and 5-HT availability) in the prefrontal cortex (PFC), dorsal striatum (dSTR), nucleus accumbens (NAcc), amygdala (AMY), and dorsal hippocampus (HPC), of C57 and DBA mice (n = 7–8 samples for each strain and brain area under 2 and 8 mpc). Two-way ANOVA for DA and 5-HT assays in each brain area is summarized in Suppl. Table S1. 5-HT availability was higher in C57 mice than DBA mice in all brain areas examined, with significant difference in the PFC and dSTR, and marginally significant difference in the AMY and HPC (Fig. 4b). In contrast, no strain difference was observed for DA availability in any region (Fig. 4e). Alterations associated with housing density were evaluated by taking the ratio of measurements under 8 mpc over 2 mpc (Fig. 4c,f). The results of one sample t-test are summarized in Suppl. Table S2. No alterations associated with housing density was observed in 5-HT, except decrease in the AMY of DBA mice under 8 mpc compared to 2 mpc (Fig. 4c). DA availability was significantly decreased in the NAcc and AMY of DBA, but not C57, mice under 8 mpc compared to 2 mpc (Fig. 4f).

These results suggest that 5-HT, but not DA, availability in C57 mice is higher than DBA mice.

### Stress associated with housing density

**Figure 5:**
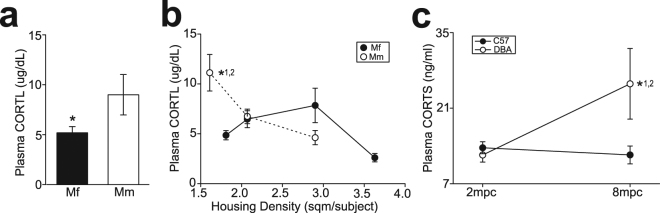
The effects of housing density on plasma stress hormones in rodents and primates. (**a**) A graph showing plasma CORTL concentrations in Japanese and rhesus macaques. *p = 0.027 for Mf vs. Mm. (**b**) A graph showing the relationships between housing density and plasma CORTL concentrations in group-housed Japanese and rhesus macaques. *^1^p < 0.001 vs. Mm2.9, *^2^p < 0.001 vs. Mf1.8. (**c**) A graph showing plasma CORTS in C57 and DBA mice under 2 mpc and 8 mpc. *^1^p = 0.0497 for 2 mpc vs. 8 mpc in DBA, *^2^p = 0.041 for C57 vs. DBA at 8 mpc.

Social crowding has been widely used as a stress model of rodents^23,24^. Difference in social interactions under high social density conditions between Japanese and rhesus macaques as well as between C57 and DBA mice might impact on stress associated with crowding differently between them. To examine this issue, we assessed plasma cortisol (CORTL) concentrations using ELISA in group-housed macaques. Plasma CORTL concentrations were significantly higher in rhesus than Japanese macaques (unpaired t-test, t_83_ = 2.247, p = 0.027; Fig. 5a). When housing density was considered in relation to plasma CORTL concentrations, plasma CORTL concentrations of rhesus macaques housed at the highest housing density (1.6 m^2^/subject) were significantly higher than those living in lower housing density (2.9 m^2^/subject; one-way ANOVA, F_6,51_ = 6.357, p < 0.001; p < 0.001 in Mm2.9 vs. Mm1.6; Fig. 5b) as well as those of Japanese macaques at the highest housing density (1.8 m^2^/subject; p < 0.001 in Mf1.8 vs. Mm1.6; Fig. 5b).

We further examined plasma corticosterone (CORTS) concentrations in C57 and DBA mice under 2 mpc and 8 mpc. Four out of 8 mice were randomly picked up from 2 cages (n = 8 for each of C57 and DBA under 8 mpc), and one of 2 mice were randomly picked up from 8 cages (n = 8 for each of C57 and DBA under 2 mpc). Two-way ANOVA revealed significant difference in the interaction between strain and housing density (F_1,26_ = 3.25, p = 0.083 for strain; F_1,26_ = 3.26, p = 0.083 for housing density; F_1,26_ = 4.88, p = 0.036 for interaction). Significantly higher CORTS was observed in DBA mice under 8 mpc than that under 2 mpc (p = 0.0497; Fig. 5c) and C57 mice under 8 mpc (p = 0.041; Fig. 5c).

These results suggest that higher social density is more stressful in primates and rodents with lower 5-HT concentrations than those with higher 5-HT concentrations.

### Anxiety

**Figure 6:**
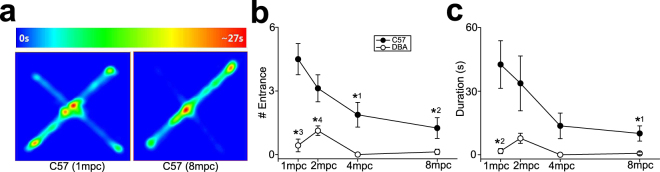
The effects of housing density on anxiety of mice. (**a**) Examples of heat maps for time and locations that C57 mice under 1 mpc and 8 mpc conditions spent in the elevated plus maze test. (**b**) A graph showing a number of opened arm entrance in the elevated plus maze. *^1^p = 0.004 for 1 mpc vs. 4 mpc in C57, *^2^p < 0.001 for 1 mpc vs. 8 mpc in C57, *^3^p < 0.001 for C57 vs. DBA under 1 mpc, *^4^p = 0.038 for C57 vs. DBA under 2 mpc. (**c**) A graph similar to (**b**), but showing duration that mice stayed in the opened arms. *^1^p = 0.023 for 1 mpc vs. 8 mpc in C57, *^2^p = 0.002 for C57 vs. DBA under 1 mpc.

Difference of affective states such as anxiety, rather than social factors such as more frequent fighting, might cause different stress responses between DBA and C57 mice under social crowding. This possibility was examined with elevated plus maze test (n = 7–8 in each strain and each housing density; Fig. 6a).

Significant difference in the number of opened arm entrance was observed between C57 and DBA mice, along with the effects of housing density (two-way ANOVA, F_1,55_ = 47.8, p < 0.001 for strain; F_3,55_ = 7.06, p < 0.001 for housing density; F_3,55_ = 3.31, p = 0.027 for interaction; Fig. 6b). C57 mice exhibited the highest number of entrance into the opened arms when they were housed alone (1 mpc), and this was decreased as housing density increased (p = 0.004 for 1 mpc vs. 4 mpc; p < 0.001 for 1 mpc vs. 8 mpc; Fig. 6b). In contrast, no alteration was observed in DBA mice with housing density, which might be due to the ceiling effects, as the number of entrance was already very low even under 1 mpc in this strain (Fig. 6b). This resulted in significantly higher number of opened arm entrance in C57 mice than DBA mice under 1 mpc (p < 0.001) and 2 mpc (p = 0.038), but not 4 mpc and 8 mpc (Fig. 6b). A similar pattern of strain difference and the effects of housing density were observed in duration that mice stayed in the opened arms (F_1,55_ = 22.3, p < 0.001 for strain; F_3,55_ = 3.48, p = 0.022 for housing density; F_3,55_ = 2.16, p = 0.103 for interaction; p = 0.023 for 1 mpc vs. 8 mpc in C57; p = 0.062 for 1 mpc vs. 4 mpc in C57; p = 0.002 for C57 vs. DBA at 1 mpc; Fig. 6c).

These results suggest that crowding heightens anxiety in C57 mice, and thereby, such heightened anxiety does not explain lower stress level in C57 than DBA mice in crowding. Moreover, alterations of anxiety across different housing density are also not associated with stress, since social isolation, which has also been widely used rodent stress model^1^, did not heighten anxiety in C57 mice. Collectively, these results suggest that stress associated with crowding may be due to social factors.

### Effects of decreasing 5-HT availability in rodents

**Figure 7:**
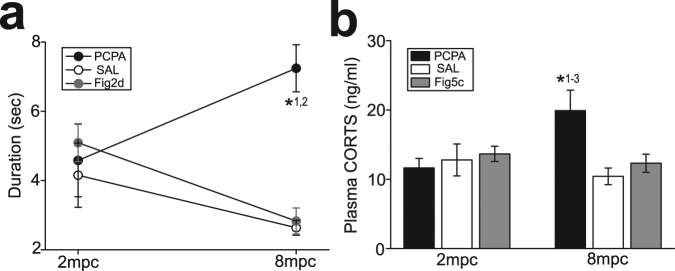
The effects of decreasing 5-HT availability on social interactions and stress hormones in C57 mice. (**a**) A graph showing duration of social interactions in mice with PCPA and control (SAL) treatments under 2 mpc and 8 mpc, and the data of C57 mice shown in Fig. 2d. *^1^p = 0.019 for PCPA vs. SAL under 8 mpc, *^2^p = 0.009 for PCPA vs. Figure 2d under 8 mpc. (**b**) A graph showing plasma CORTS concentrations in mice with PCPA and SAL treatments under 2 mpc and 8 mpc, and the data of C57 mice shown in Fig. 5c. *^1^p = 0.006 for PCPA vs. SAL under 8 mpc, *^2^p = 0.012 for PCPA vs. Figure 5c under 8 mpc, *^3^p = 0.042 for PCPA under 2 mpc vs. 8 mpc.

Although the above observations suggest that 5-HT level may be associated with decreased social interactions, which mitigate stress, in crowding, a causal relationship between them remained unclear. Thus, we further investigated the effects of decreasing 5-HT availability by administration of the tryptophan hydroxylase inhibitor, parachlorophenylalanine (PCPA; 300 mg/kg, i.p.) in C57 mice under 2 mpc and 8 mpc. Duration of social interactions in mice with PCPA under 8 mpc were significantly longer than those receiving saline (SAL) treatments and shown in Fig. 2d under 8 mpc, whereas such alterations with PCPA administration was not observed in mice under 2 mpc (two-way ANOVA, F_1,114_ = 0.010, p = 0.920 for density; F_2,114_ = 3.12, p = 0.048 for drug treatments; F_2,114_ = 3.52, p = 0.033 for interaction; p = 0.019 for PCPA vs. SAL, p = 0.009 for PCPA vs. Figs 2d, 7a). Moreover, plasma CORTS in mice with PCPA under 8 mpc, but not under 2 mpc, was significantly higher than those with control treatments and shown in Fig. 5c under 8 mpc (two-way ANOVA, F_1,37_ = 0.98, p = 0.327 for density; F_2,37_ = 2.51, p = 0.095 for drug treatments; F_2,37_ = 4.82, p = 0.013 for interaction; p = 0.006 for PCPA vs. SAL, p = 0.012 for PCPA vs. Figs 5c, 7b). Moreover, plasma CORTS in C57 mice with PCPA under 8 mpc was also significantly higher than mice with SAL treatments under 8 mpc (p = 0.006; Fig. 7b) and mice with PCPA under 2 mpc (p = 0.042; Fig. 7b).

These results suggest that 5-HT availability itself may not affect social interactions, as social interactions of mice under 2 mpc was not altered by PCPA administration. However, high 5-HT availability may be a prerequisite that causes decreased social interactions, along with mitigation of stress, in crowding.

## Discussion

In this study, we found that Japanese macaques exhibited higher plasma 5-HT concentrations than rhesus macaques. Similarly, C57 mice exhibited higher plasma 5-HT concentrations and higher 5-HT availability in mesocorticolimbic regions than DBA mice. In both rodents and primates, animals with higher 5-HT concentrations (hyperserotonemia) exhibited lower social interactions especially when they were housed in crowding. Since these observations are correlative, we also examined the effects of PCPA administration in C57 mice, further supporting that hyperserotonemia was at least partly causing housing density-dependent social interaction alterations. In addition, the striking finding is that, although stress hormone was elevated in DBA mice and rhesus macaques under crowding, this was not observed in C57 mice and Japanese macaques, suggesting that behavioral alterations such as social avoidance are not consequence of stress, but may be an adaptive behavioral strategy to mitigate stress in adverse social environments.

There are several limitations in the current study. First, social interactions in rodents were assessed only as distance between pairs of mice, such that the current analysis did not separate types of social interactions. Another limitation would be the correlative nature of the study. This issue was partly overcome by investigating the effects of PCPA administration in rodents, which provided the link between behavioral and neurochemical data. However, it is also highly unlikely that 5-HT is the only molecule involved in housing density effects on social interactions. Indeed, we found that hyperserotonemia would be just a prerequisite for decrease of social interactions under crowding, and it did not affect social interactions by itself. In contrast, we also found that DA and 5-HT metabolites were also altered in non-stressed C57 mice under crowding (Suppl. Results, Suppl. Figure S1), suggesting that DA and 5-HT synaptic transmission may also be involved in expressions of decreased social interactions under crowding. Therefore, further explorations of other molecules and mechanisms involved in the housing density effects are important issues to be addressed in future studies. In this study, we also examined only two strains of mice and two species of primates, since the aim of this study was to explore whether monoamines were involved in housing density effects, but not to identify which strains of mice or species of primates exhibit alterations of social interactions by crowding. Nonetheless, our study has demonstrated that similar housing density effects can be observed even “across species” in both rodents and primates, although these may not be identical between them, suggesting that it is unlikely that crowding effects take place only in one strain of mice or one species of primates.

There is a number of studies investigating the effects of crowding in human subjects. However, the findings in these studies are inconsistent, which is thought to be due to various factors^27–32^. The effects of housing density have also been investigated in non-human primates. In chimpanzees, crowding decrease social interactions such as aggressions to others, along with no clear stress associated with such crowding^5^. Capuchins are found similar, decreased social interactions and aggression in crowded environments, and no stress^6^. In contrast, rhesus macaques exhibit even increases of social affiliations without alterations of aggressions to others^7^. Moreover, different from chimpanzees, crowding increases stress hormones in rhesus macaques^33^. Our current findings in rodents and primates are mostly consistent with these previous studies, along with providing a new insight that such housing density-dependent alterations of social behaviors may be associated with hyperserotonemia. However, hyperserotonemia itself may not be a causal factor of such social behavioral changes, as it presents in animals regardless of housing density. On the other hand, hyperserotonemia appears to be a prerequisite for such housing density-dependent social behavioral changes. One possible explanation for such observation may be cognitive overloads by crowding^4^, given the association between hyperserotonemia and sensory hyper-sensitivity^34^. Indeed, C57 mice have been shown more sensitive to sensory stimuli than DBA mice^26^, although whether sensory sensitivity may be different between Japanese and rhesus macaques have remained unknown.

We found that anxiety became stronger when housing density was increased in C57 mice, whereas stress hormone was not elevated in this strain under crowding. This discrepancy of housing density effects between anxiety and stress hormone in C57 mice suggests that stress caused under crowding is not due to anxiety, but other social factors, such as more frequent fighting and social defeats, something specifically associated with crowding conditions. In addition, no elevation of stress hormone in C57 mice under crowding suggests that, although crowding has been widely used as a stress model in mice, such crowding stress model is not always appropriate for all strains of mice. For instance, consistent with our study, there is a study showing that stress level of C57 mice is even lower when they are in isolated housing than group housing^35^, even though social isolation, just like social crowding, has also been widely used as a stress model in mice. Thus, C57 mice appear to be more resistant to not only social crowding, but also social isolation, which is articulated by the observation in the current study that C57 mice exhibited low social interactions.

We also found several additional important issues in this study. First, although the effects of housing density on social interactions were similar between rodents and primates, social interactions in C57 mice was lower than that in DBA mice even under dispersing social groups, whereas social interactions of Japanese macaques in low housing density conditions were comparable to those of rhesus macaques. The reason for such difference has remained unclear. We also observed decreases of 5-HT availability in the AMY and DA availability in the NAcc and AMY of DBA mice under crowding. Such alterations may most likely be a consequence of stress induced by crowding, as DBA, but C57, mice exhibited stress elevation under crowding. Consistent with it, we found that amphetamine-induced locomotion was not different across different housing density in C57 mice, whereas inverted U-shape responses in relation to housing density was observed in DBA mice (Suppl. Results, Suppl. Fig. S2). In addition, a study by Calhoun has demonstrated that DBA mice create smaller social groups than C57 mice when they are housed in a large room with food and water available ad libitum^36^. This is consistent with our finding, as DBA mice exhibited higher stress level than C57 mice at the same housing density. In order to maintain larger social groups, establishment of more rigid social hierarchy within the groups would be also crucial. We found that social hierarchy of both DBA and C57 mice became less clear as housing density increased, with DBA mice exhibiting greater vulnerability than C57 mice (Suppl. Results, Suppl. Fig. S3), partly consistent with the studies showing that 5-HT transmission plays important roles in social hierarchy as well^37,38^.

Hyperserotonemia^39,40^, sensory hyper-sensitivity^41^, and social impairments^42^, are consistently observed in ASD. A recent genetic study has demonstrated that ASD individuals with the allele that causes hyperserotonemia exhibit stronger sensory hyper-responses^34^. Moreover, transgenic mice engineered to enhance 5-HT reuptake, which thereby decrease synaptic transmission, but may also increase tissue concentrations of 5-HT, exhibit increased plasma 5-HT concentrations and impaired social interaction with mates^43^. Although highly speculative, our study suggests that social density may be an important factor for understanding of these attributes in ASD. This notion may also be associated with increasing prevalence of ASD over the past decades^44^, which appears to take place primarily in urban areas^45^. Epidemiological studies have also demonstrated that offspring born from parents at older ages has a igher risk of ASD, along with increases of *de novo* mutations on ASD susceptible genes^46–48^. On the other hand, over-population also delays timing of reproduction in humans^49^ and animals^50–52^, along with delayed sexual maturation of females by antenatal maternal exposure to crowding environments during pregnancy^50,51^. In the Calhoun’s famous rodent utopia experiments^53^ in which the environment of enclosed room with other resources available as much as they needed was provided, explosion of population happened followed by fighting with each other with cannibalism and infanticide toward tailing off to extinction at the end in rodents, which Calhoun called “behavioral sink”. Notably, rodents that had survived at the end of experiments exhibited behaviors partly consistent with ASD such as socially withdrawals^53,54^.

In conclusion, our study suggests that 5-HT may be an important molecule associated with social behaviors, which interact with social environments such as population density. Such biological mechanisms may explain some aspects of psychiatric disorder such as ASD.

## Methods

### Subjects

All experiments were conducted in accordance with the *Science Council of Japan Guidelines for Proper Conduct of Animal Experiments* and approved by the Kyoto University Primate Research Institute Animal Experiment Committee.

For rodent experiments, male C57 (C57BL/6NCrlCrlj) and DBA (DBA/2NCrlCrlj) mice at 8 weeks old upon arrival, which were approximately equal weights and without kinship, were purchased from Charles-River Japan. Mice of the same strain were grouped into different numbers of mice per cage for at least 2 weeks before starting experiments. These mice were housed together until all experiments were completed in the temperature controlled room with a normal 12:12 hour light-dark cycle. A floor space of cages used for housing was 414 cm^2^ (18.0 × 28.0 cm), such that theoretical allocations of living space for each mouse were 207, 103.5, and 51.75 cm^2^, respectively, when they were grouped in 2, 4, and 8 mice per cage (2 mpc, 4 mpc, and 8 mpc).

For non-human primate experiments, 5 groups of Japanese macaques (*Macaca fuscata*; Mf) and 4 groups of rhesus macaques (*Macaca mulatta*; Mm) with different housing density were used for behavioral observations. All groups were housed in the cages at the same size with 14.5 m^2^ floor space. In Japanese macaques, 1 group of 4 subjects/cage (3.6 m^2^/subject), 1 group of 5 subjects/cage (2.9 m^2^/subject), 1 group of 7 subjects/cage (2.1 m^2^/subject), and 2 groups of 8 subjects/cage (1.8 m^2^/subject), were subjected for behavioral observations. In rhesus macaques, 2 groups of 5 subjects/cage (2.9 m^2^/subject), and 1 group of 7 subjects/cage (2.9 m^2^/subject) and 1 group of 9 subjects/cage (1.6 m^2^/subject), were subjected for behavioral observations. Each group consisted of a mixture male and female macaques with different ages.

### Behavioral observations in non-human primates

Behavioral observations in Japanese and rhesus macaques were conducted with the “all occurrences of some behaviors” sampling method^55^. Briefly, two observers with video cameras stood approximately 0.5–1.0 meter away from the cage during observations, and monitored and recorded specific monkey behaviors, i.e. social affiliations and aggressions, that occurred in each cage during each recording. A recording was conducted 30 minutes per day, which was divided into 3 sequences of 10 minutes each with intervals of approximately 30 minutes between the sequences. Recordings were repeated for 10 days for each cage. Presence or absence of social affiliations and aggressions in every 10 seconds (1,800 frames for 30 minutes) were measured, and a number of frames in which the events were present was expressed as percentage of time out of a whole recording period that these events occupied. Social affiliations and aggressions were defined as we have previously described^56^. Inter-rater reliabilities for the rates of aggressions and social affiliations between the experimenters at off-line analysis were very high (Cohen’s kappa = 0.93, with confidence interval at 0.90–0.96), indicating reliable behavioral assessments^57^.

### Rodent social interaction observations

Social interactions of mice were examined in their home cages, under the housing density of 2 mpc, 4 mpc, and 8 mpc. During mice were freely moving in their home cages located in the room with dim light, the cages were video recorded at 6 times, with each time of recordings for each 30 seconds, along with the 3 minutes intervals between recordings. Then, using MTrackJ plug-in of ImageJ software (https://imagescience.org/meijering/software/mtrackj/)^58^ (Fig. 3a,b), which enabled to track moving objects manually by experimenters off-line through frame-by-frame advance of video recordings, each freely moving mouse in the cages was tracked. This tracking method was chosen over other available automated tracking software, since although several automated tracking software were tried, none of them was able to track mice at sufficient precisions, especially under the crowding condition such as 8 mpc.

In this social interaction analysis, social interactions, although whether such interactions were affiliative or aggressive contacts was undistinguished, were defined as the heads of a pair of mice reached less than 5 cm (given approximate body size of a mouse at 5 cm) at any moments in the recordings. Duration that the heads of a pair of mice were less than 5 cm in every 30 second recording periods was quantified. However, it was also possible that such events happened by chance. To address this problem, tracking of a mouse in one cage was randomly paired with tracking of a mouse in another cage, and duration of the events that the heads of the randomized pairs of mice reached less than 5 cm was quantified. This random shuffling of pairs was repeated for 500 times, and the threshold of duration (*Tm*) for social interactions that was longer than the chance level (higher than 2 standard deviations from the mean of duration in the randomized pairs, or p < 0.05) was determined. Thus, duration of events observed in any pairs of mice that were longer than *Tm* were considered as meaningful social interactions. In addition, using the tracking data, a probability that a mouse was found in specific location of the cage was measured, and Shanon’s information theory was applied to assess mutual information for social interactions in a pair of mice as well as that a whole group^59^. To calculate mutual information, first, a cage was divided into 17 × 11 areas, and probability, p(x_i_), p(x_j_), p(x_k_)…, that mice i, j, k… were found in each area (percentages of time that the mice spent in each area during a whole observation period) in the cages were calculated (Fig. 2g). Then, the uncertainty of the location of mouse i in the cage was expressed as the entropy H(x_i_) = −Σp(x_i_)log_2_p(x_i_), and the uncertainty of the location of the mouse i given by the locations of other mice j, k… was expressed as the joint entropy H(x_i_|{x_j_,x_k_,x_l_…}) = −Σp(x_i_,{x_j_,x_k_,x_l_…})log_2_p(x_i_|{x_j_,x_k_,x_l_…}). Accordingly, the mutual information between the location of mouse i and those of mice j, k… was given by I(x_i_;{x_j_,x_k_,x_l_…}) = H(x_i_) − H(x_i_|{x_j_,x_k_,x_l_…}).

### Three-chamber sociability test in rodents

To further evaluate the effects of housing density on social interactions of mice, motivation to socially interact with mates was assessed in mice under 2 mpc and 8 mpc using three-chamber sociability test, as we have previously conducted^60^. In this test, a mouse was placed in the middle of three partitioned chamber, with the middle chamber connected to two other chambers on each side. In one of the two chambers, a mouse of the same strain as the mouse being tested and that had no previous contact was caged. Only a mesh cage without a mouse was placed in the other side of the chamber. The amounts of time that the tested mouse spent in each side of the chamber were measured for 10 minutes. Ratio of time spent in the social over non-social chambers (S/NS ratio) was calculated.

### Elevated plus maze test

Elevated plus maze test was conducted to examine anxiety of mice under different housing density (1, 2, 4 and 8 mpc), as we have previously conducted^60^. The maze consisting of 2 opened arms facing each other and 2 closed arms by the walls that were located perpendicular to the opened arms was used. These arms were elevated 1 m above from the ground. A mouse was placed in the central arena, and allowed to freely explore the maze for 10 minutes. The amounts of time that mice spent in the opened arms and the number of entrance into the opened arms were measured.

### HPLC

In rodents, high-performance liquid chromatography (HPLC) was conducted to investigate tissue DA and 5-HT concentrations (DA and 5-HT availability) in corticolimbic brain areas. After completion of all behavioral tests, mice under 2 mpc and 8 mpc were anesthetized by administration of the over-dose of pentobarbital (100 mg/kg, i.p.), and decapitated. Brains were removed from the skulls, and frozen immediately by a medical freeze spray. Brains were then sectioned and tissues of the target areas were sampled using tissue preparation cutting tips. Samples were obtained from the prefrontal cortex (PFC; prelimbic and infralimbic cortex combined), dorsal striatum (dSTR; dorsomedial part), nucleus accumbens (NAcc; core and shell regions combined), amygdala (AMY; basolateral and lateral nuclei combined), and hippocampus (HPC; dorsal CA1). Tissues obtained from the left and right hemispheres were combined for subsequent processing. A tissue sample from each region was placed in 490 μL of ice cold 0.2 M percloric acid, and isoproterenol (ISO; 100 ng in 10 μL solution) was added as an internal standard. Samples were homogenized using an ultrasonic homoginizer, and left on ice for 30 minutes. For later, separate protein assays, 10 μL was aliquoted from each sample before centrifuge. Samples were centrifuged at 14,500 rpm for 15 min. Supernatants were then aliquoted, and 10 μL of each sample was applied for HPLC. Protein assays of samples were conducted using Protein Assay Rapid Kit (WAKO, Osaka, Japan) according to the manufacturer’s instruction, and 96-well microplates were read using the microplate reader.

In addition to brain tissues, HPLC was also conducted to examine plasma DA concentrations in primates and rodents, and plasma 5-HT concentrations of rodents. For these assays, plasma samples were centrifuged at 10,000 rpm for 5 minutes. Then, 100 μL each of supernatants was taken. Processing of further purification of DA molecules was conducted using the Clean Column EG kit from Eicom, along with addition of 10 μL of 10 pg/μL internal standard (ISO) into the samples. For plasma 5-HT concentrations in rodents, 100 μL of 0.5 mol/L perchloric acid and ISO were added into plasma samples, and centrifuged again at 2,000 rpm for 15 minutes. Ten micro-litter each of supernatants from these samples was applied for HPLC.

Using a similar procedure described in our previous study^61^, HPLC was carried out using a HTEC-500 HPLC electrochemical detection system (Eicom, Tokyo, Japan) with EICOMPAK SC-5ODS column and CA-ODS pre-column. For rodent brain samples, the mobile phase consisting of 0.1 M acetic acid-citric acid buffer (pH 3.5) and methanol (83:17, v/v), along with 190 mg/L sodium 1-octanesulfonate and 5 mg/L EDTA-2Na, was used. For non-human primate and rodent plasma DA samples, the mobile phase consisting of 0.1 mol/L phosphate buffer solution (pH5.7) and methanol (88:12, v/v), along with 450 mg/L sodium 1-octanesulfonate and 50 mg/L ethylenediaminetetraacetic acid (EDTA) disodium salt dihydrate, was used. For rodent plasma 5-HT samples, the mobile phase consisting of 0.1 mol/L phosphate buffer solution (pH6.0) and methanol (80:20, v/v), along with 400 mg/L sodium 1-octanesulfonate, and 50 mg/L EDTA-2Na, was used. The mobile phase flow rate was 500 μL/min, and the working electrode was set at either +400 (for plasma 5-HT samples) or 750 mV (plasma DA and brain samples) vs. Ag/AgCl reference electrode. The standard with the known concentration of DA, 5-HT, and ISO, were used to quantify and identify the peaks on the chromatographs. Quantification of DA and 5-HT in samples was based on the following formula: (*ISO*_*std*_*/TA*_*std*_) *x* (*TA*_*spl*_*/ISO*_*spl*_) *x A x* (*1/B*), where *ISO*_*std*_ and *TA*_*std*_ are the areas of the peaks of ISO and a target substance (DA or 5-HT) in the standard; *ISO*_*spl*_ and *TA*_*spl*_ are the areas of the peaks of ISO and a target substance in a sample; *A* is the amount of ISO added to the sample; and *B* is the amount of tissue proteins in the sample.

### ELISA

Plasma corticosterone (CORTS) in mice under 2 mpc and 8 mpc and was assessed with enzyme-linked immunosorbent assay (ELISA). Blood samples were collected in parallel with decapitation of mice for sampling of brains to be used in HPLC. All blood samples were collected between 10:00 am and 12:00 pm of the days. A blood sample of approximately 1 mL from each mouse was collected into a heparinized microcentrifuge tube. The tubes were centrifuged at 3,000 rpm for 15 minutes. Plasma was then aliquoted and stored in the deep freezer until assays. Samples were processed using the ELISA kit for CORTS (Enzo Life Science, Farmingdale, NY; Catalog #ADI-900-097) according to the manufacturer’s instruction.

ELISA was also conducted to assess plasma concentrations of cortisol (CORTL) and 5-HT in group-housed Japanese and rhesus macaques. Approximately 1–2 mL of a blood sample from each monkey was obtained with a heparin-coated syringe under anesthesia. Samples were then centrifuged at 3,000 rpm for 5 minutes, and plasma were obtained and stored in the freezer until the days of processing for ELISA. Plasma were further processed according to the manuals of the ELISA assay kits from Salimetrics (Catalog #1–3002) and Arigo Biolaboratories (Catalog #ARG80480) for CORT and 5-HT, respectively.

After processing, ELISA plates were read using the iMark microplate reader (Bio-rad, Hercules, CA).

### PCPA administration

The tryptophan hydroxylase inhibitor, parachlorophenylalanine (PCPA) was purchased from Tocris Bioscience. PCPA was dissolved in 0.3 ml 0.9% saline at the dose of 300 mg/kg, and intraperitoneally administered in C57 mice under 2 mpc and 8 mpc. The drug was administered in all mice in each cage. An equivalent volume of saline (SAL) was given to mice as control treatments. Recordings of social interactions in home cages were conducted 24 hours after drug administration, followed by sacrificing animals under 2 mpc and 8 mpc, with blood sampling for CORTS assays. The dose of the drug and timing of behavioral observations after drug administration were determined based on the previous study showing that PCPA at this dose substantially reduced tryptophan hydroxylase expression up to 5 days after administration^62–64^.

### Data analysis

Data collection and statistical analyses were conducted by investigators who were not blinded to the experimental conditions. No data points were removed from statistical analysis. Sample sizes were not predetermined by statistical methods. All data analyses were conducted off-line. Statistical analysis was conducted using Origin Pro ver9.0 and Statistica ver7.0 software. A probability value of p < 0.05 was considered to indicate statistical significance.

### Data availability

The datasets generated and analysed during the current study are available from the corresponding author on reasonable request.

## Electronic supplementary material

Supplementary Information
