# Supplementary material for: The Effects of Housing Density on Social Interactions and Their Correlations with Serotonin in Rodents and Primates
Source: Sci Rep. 2018 Feb 22;8:3497. doi: 10.1038/s41598-018-21353-6 (PMC5823940; doi:10.1038/s41598-018-21353-6)
Supplement: Supplementary file 1 — Supplementary Information [file 41598_2018_21353_MOESM1_ESM.pdf]

Supplementary Information

The Effects of Housing Density on Social Interactions and Their Correlations with Serotonin in Rodents and Primates

Y.-A. Lee, T. Obora, L. Bondonny, A. Toniolo, J. Mivielle, Y. Yamaguchi, A. Kato, M. Takita, & Y. Goto

Supplementary Results

Supplementary Methods

Supplementary References

Supplementary Table S1

Supplementary Table S2

Supplementary Table S3

Supplementary Table S4

Supplementary Table S5

Supplementary Figure S1

Supplementary Figure S2

Supplementary Figure S3

Supplementary Results

Monoamine metabolites in rodents

In addition to tissue DA and 5-HT concentrations, we also examined ratios of 3,4-dihydroxyphenylacetic acid (DOPAC)/DA, homovanillic acid (HVA)/DA, and 5-hydroxyindoleacetic acid (5-HIAA)/5-HT, as DA and 5-HT turnovers with synaptic release have been shown correlated with these metabolites, and thereby, the ratios between monoamines and their metabolites can be utilized as index reflecting the amounts of synaptic release of these monoamines [1-3].

Two-way ANOVA for each of DOPAC/DA, HVA/DA, and 5-HIAA/5-HT, in each brain area is summarized in Suppl. Table S3. DOPAC/DA was significantly higher in the corticostriatal pathways of DBA mice than those of C57 mice (Suppl. Table S3). Alterations associated with housing density were evaluated by taking the ratios of those measurements under 8mpc over 2mpc (Fig. 1d-f), and the results of one sample t-test are summarized in Suppl. Table S4. DOPAC/DA was significantly decreased in the PFC of C57 mice under 8mpc compared to 2mpc (Suppl. Table S4; Fig. 1d). HVA/DA was also decreased in the PFC of both C57 and DBA mice (Table S4; Fig. 1e), along with decreases in the NAcc and HPC of C57BL mice selectively (Table S4; Fig. 1e). These alterations selectively observed in C57 mice, or commonly observed in both strains, but more prominent in C57 than DBA mice, may be associated behavioral changes that mitigate crowding stress in C57 mice.

These results suggest that DA and 5-HT transmission may be involved in expression of decreased social interactions in mice under crowding social groups.

Novelty- and DA-dependent locomotion

The effects of social crowding were investigated in locomotor activity induced by novel environment exposure and cocaine administration (Suppl. Methods, Suppl. Fig. S2a, b).

In novelty-dependent (spontaneous) locomotion, mice were housed alone (1mpc), 2mpc, 4mpc, or 8mpc. Seven to 8 mice from each housing condition (one mice per cage at 1mpc and 2mpc; two out of 4 mice per cage at 4mpc; four out of 8 mice at 8mpc) were subjected for tests. Mice were placed in the open field chamber that had not been exposed before, and locomotor distance that they travelled was measured for 10 minutes. Two-way ANOVA revealed significant effects of housing density ($F_{3,56}$=12.1, $p<0.001$; Suppl. Fig. S2c), but no difference in strain ($F_{1,56}$=2.43, p=0.124) and their interaction ($F_{3,56}$=0.462, p=0.710). Significantly higher locomotor distance was observed in C57 mice at 8mpc than 1mpc (p=0.012) and in DBA mice at 8mpc than 1mpc (p=0.009) and 2mpc (p=0.009; Suppl. Fig. S2c).

In addition, DA-dependent locomotion was examined with acute cocaine administration (15 mg/kg, i.p.; Suppl. Fig. S2a, b). Significant difference in locomotor responses to cocaine administration was observed in strain ($F_{1,56}$=4.24, p=0.044; Suppl. Fig. S2d) and housing density ($F_{3,56}$=3.82, p=0.015; Suppl. Fig. S2d), but not in their interaction ($F_{3,56}$=1.83, p=0.152). DBA mice at 4mpc exhibited significantly or marginally significantly higher cocaine-induced locomotion than those at 1mpc (p=0.007) and 2mpc (p=0.086), which resulted in inverted U shape-like relationship between cocaine-induced locomotion and housing density (Suppl. Fig. S2d). In contrast, housing density did not alter cocaine-induced locomotion in C57 mice (Suppl. Fig. S2d), with which, although not statistically significant, cocaine-induced locomotion

was higher in DBA mice than C57 mice at 4mpc ($p=0.077$; Suppl. Fig. S2d).

These results suggest that mice under crowding environments are more locomotive than those in dispersing environments.

Social hierarchy

We further investigated the effects of crowding on group structures such as social hierarchy using tube rank test (Suppl. Methods). First, 9 groups of C57 and 10 groups of DBA mice, respectively, at 4mpc were examined. Five trials of tube rank test demonstrated stable social hierarchy in both C57 and DBA groups, although hierarchy in DBA groups were less stable than that in C57 groups (Suppl. Fig. S3a). Normalized David's score (DS) [4] as well as linearity, steepness, and stability of hierarchy were calculated based on the number of wins/loses of each subject in the test. Normalized DS was significantly lower at the lowest (4th) rank of C57 mice than the same rank of DBA mice (Mann-Whitney U test, $Z=-2.60$, $p=0.009$; Suppl. Fig. S3b), but not in other ranks (1st rank, $Z=1.60$, $p=0.110$; 2nd rank, $Z=0.88$, $p=0.379$; 3rd rank, $Z=-0.33$, $P=0.739$; Suppl. Fig. S3b). Landau's corrected index (h'), which has been used to express linearity of social hierarchy [5], tended to be lower in DBA groups than that in C57 groups ($Z=1.74$, $p=0.081$; Suppl. Fig. S3c). Steepness, which is a slope of linear regression applied to the relationship between social rank and normalized DS [6], and indicates robustness of hierarchy, was significantly lower in DBA groups than that in C57 groups ($Z=2.29$, $p=0.022$; Suppl. Fig. S3c). In addition, stability, which indicates how frequently social rank of individuals is replaced [7], was also significantly lower in DBA groups than that in C57 groups ($Z=3.30$, $p<0.001$; Suppl. Fig. S3c). A positive correlation, when the data from DBA and C57 groups were combined, was observed between steepness and stability ($r=0.672$, $p=0.002$; Suppl. Fig. S3d).

We then investigated how social hierarchy was changed by altering housing density. Social hierarchy at 3mpc (n=5 groups for each of DBA and C57 mice) and 6mpc (n=5 groups for each of DBA and C57 mice) were assessed and compared with 4mpc. Linearity, steepness, and stability of social hierarchy were the strongest at 3mpc in both DBA and C57 mice, and these became deteriorated as housing density was increased into 4mpc and 6mpc (Suppl. Fig. S3e). In addition, such deterioration of social hierarchy with housing density increases was stronger in DBA than C57 groups (Suppl. Fig. S3e). Two-way ANOVA has revealed significant difference between DBA and C57 (strain; $F_{1,33}=5.14$, $p=0.030$), but not housing density ($F_{2,33}=2.57$, $p=0.092$) or their interaction ($F_{2,33}=1.18$, $p=0.321$) in Landau's corrected index for linearity. In steepness, significant difference was found in strain ($F_{1,33}=14.8$, $p<0.001$) and housing density ($F_{2,33}=23.6$, $p<0.001$), but not their interaction ($F_{2,33}=2.92$, $p=0.068$), although it was marginally significant. Significant difference was also found in all of strain ($F_{1,33}=28.4$, $p<0.001$), housing density ($F_{2,33}=33.6$, $p<0.001$), and their interaction ($F_{2,33}=5.25$, $p=0.011$) in stability.

These results suggest that rigidness of social hierarchy in mouse social groups depend on housing density. Moreover, susceptibility of social hierarchy against housing density is higher in DBA mice than C57 mice.

Supplementary Methods

Locomotion test

Spontaneous locomotion in a novel environment and cocaine-induced locomotion were examined to evaluate novelty and DA agonist responses in mice with different housing density (1, 2, 4 and 8mpc), as we have previously conducted [8]. The test consisted of two phases. On the first day, mice, which had not been exposed the chamber before, were placed in an open field chamber, and the horizontal distance travelled in 10 minutes were measured. Then, on the second day, 15 mg/kg of cocaine, the dose determined based on other studies [9,10], was given to mice intraperitoneally 5 minutes before they were placed in the open field chamber. The horizontal distance travelled in 10 minutes were measured again.

Tube rank test

Social hierarchy of groups was determined using the tube rank test, as we have previously conducted [11,12]. Social hierarchy was examined in groups of 3, 4, and 6mpc. Briefly, an apparatus consisting of a transparent tube connected to transparent boxes at each end was used. In the middle of the tube, there was a slit to insert a transparent wall for a partition. Once mice were trained to go across the tube from one end to the other, pairs of mice from the same groups were taken and simultaneously placed with one mouse into each of the boxes on either side of the tube. When mice from each side reached the middle of the tube, the partition wall was removed. The mouse that caused the other to retreat was designated as the "winner", and the mouse that was retreated out of the tube was designated as the "loser". If no movement of either mouse was observed after 5 minutes, both mice were removed from the tube, and the trial was designated as a "draw". Tournaments of all possible combinations of matches by pairs of mice in each group were conducted once per day for 5 days (5 trials). A win or a loss was scored by +1 or 0, respectively. These scores were used to calculate ranking of subjects in the groups by Inconsistencies and Strength of Inconsistencies (I&SI) method [13]. Then, sociomatrices derived from I&SI method were used to calculate social dominance of subjects by normalized David's score (DS) [4] as well as linearity, steepness, and stability, of social hierarchy, with linearity assessed using Landau's corrected index (h') [5], steepness measured as a slope of linear regression applied to the relationships between social rank and normalized DS of subjects in the groups [6], and stability (s) calculated using the method by Perez-Escudero and colleagues [7].

Supplementary References

1 Fuller, R. W. & Perry, K. W. Effect of lergotrile on 3,4-dihydroxyphenylacetic acid (DOPAC) concentration and dopamine turnover in rat brain. *J Neural Transm* **42**, 23-35 (1978).

2 Mignot, E. & Laude, D. Study of dopamine turnover by monitoring the decline of dopamine metabolites in rat CSF after alpha-methyl-p-tyrosine. *J Neurochem* **45**, 1527-1533 (1985).

3 Johnston, C. A. & Moore, K. E. Measurement of 5-hydroxytryptamine synthesis and metabolism in selected discrete regions of the rat brain using high performance liquid chromatography and electrochemical detection: pharmacological manipulations. *J Neural Transm* **57**, 49-63 (1983).

4 David, H. A. Ranking from unbalanced paired-comparison data. *Biometrika* **74** (1987).

5 de Vries, H. An improved test of linearity in dominance hierarchies containing unknown or tied relationships. *Anim Behav* **50**, 1375-1389 (1995).

6 De Vries, H., Stevens, J. M. G. & Vervaecke, H. Measuring and testing the steepness of dominance hierarchies. *Anim Behav* **71**, 585-592 (2006).

7 Perez-Escudero, A., Vicente-Page, J., Hinz, R. C., Arganda, S. & de Polavieja, G. G. idTracker: tracking individuals in a group by automatic identification of unmarked animals. *Na Method* **11**, 743-748, doi:10.1038/nmeth.2994 (2014).

8 Lee, Y. A., Kim, Y. J. & Goto, Y. Cognitive and affective alterations by prenatal and postnatal stress interaction. *Physiol Behav* **165**, 146-153, doi:10.1016/j.physbeh.2016.07.014 (2016).

9 Jackson, H. C., Griffin, I. J. & Nutt, D. J. Buprenorphine-cocaine interactions in mice: effect on locomotor activity and hole-dipping behaviour. *J Pharm Pharmacol* **45**, 636-640 (1993).

10 Reith, M. E., Wiener, H. L. & Fischette, C. T. Sertraline and cocaine-induced locomotion in mice. I. Acute studies. *Psychopharmacol (Berl)* **103**, 297-305 (1991).

11 Yamaguchi, Y., Lee, Y. A., Kato, A. & Goto, Y. The roles of dopamine D1 receptor on the social hierarchy of rodents and non-human primates. *Int J Neuropsychopharmacol* **20**, 324-335, doi:10.1093/ijnp/pyw106 (2017).

12 Yamaguchi, Y., Lee, Y. A., Kato, A., Jas, E. & Goto, Y. The Roles of Dopamine D2 Receptor in the Social Hierarchy of Rodents and Primates. *Sci Rep* **7**, 43348, doi:10.1038/srep43348 (2017).

13 De Vries, H. & Appleby, M. C. Finding an appropriate order for a hierarchy: a comparison of the I&SI and the BBS methods. *Anim Behav* **59**, 239-245, doi:10.1006/anbe.1999.1299 (2000).

Table S1. A summary of statistical analysis for comparisons of plasma monoamine and stress hormone concentrations in macaque groups with the same housing density.

	Mean ± s.e.m.		*Unpaired t-test*	
**Japanese macaque**				
	Group 1 (1.8 m^2/sub)	Group 2 (1.8 m^2/sub)		
5-HT (ng/mL)	386.3 ± 88.6	469.1 ± 110.2	t_{14}=0.585	p=0.568
DA (pg/μL)	169.9 ± 56.3	94.1 ± 37.8	t_{14}=1.12	p=0.282
CORTL (μg/dL)	4.82 ± 0.67	4.89 ± 0.69	t_{14}=0.072	p=0.943
**Rhesus macaque**				
	Group 1 (2.9 m^2/sub)	Group 2 (2.9 m^2/sub)		
5-HT	28.7 ± 13.9	25.7 ± 8.27	t_8=0.186	p=0.857
DA	246.5 ± 43.5	222.2 ± 33.2	t_8=0.444	p=0.669
CORTL (μg/dL)	5.26 ± 1.29	3.98 ± 0.65	t_8=0.886	p=0.402

Table S2. A summary of two-way ANOVA in HPLC assays of monoamines.

		Strain		Density		Interaction	
		$F_{1,27}$	*p value*	$F_{1,27}$	*p value*	$F_{1,27}$	*p value*
**DA**	*PFC*	0.258	0.615	0.835	0.369	1.89	0.181
	dSTR	0.001	0.998	0.744	0.396	0.172	0.682
	NAcc	0.005	0.947	3.20	0.085	0.752	0.393
	AMY	1.12	0.300	3.01	0.094	0.211	0.650
	HPC	7.08	0.013*	0.546	0.466	0.281	0.600
**5-HT**	*PFC*	19.4	<0.001*	0.029	0.866	0.395	0.535
	dSTR	14.1	<0.001*	0.107	0.746	0.525	0.475
	NAcc	1.48	0.235	0.070	0.793	0.107	0.746
	AMY	3.82	0.061	3.00	0.094	0.396	0.535
	HPC	3.64	0.067	0.121	0.731	0.293	0.593

*: Statistical significance

Table S3. A summary of one sample t-test for the ratios of monoamines in mice at under 8mpc over 2mpc (8mpc/2mpc).

		**C57**		**DBA**	
		t_7	*p value*	t_7	*p value*
**DA**	*PFC*	-0.729	0.489	1.92	0.096
	dSTR	-0.800	0.450	-1.03	0.337
	NAcc	-1.89	0.101	-6.72	<0.001*
	AMY	-2.09	0.075	-13.2	<0.001*
	HPC	-1.13	0.300	-0.340	0.744
**5-HT**	*PFC*	0.561	0.592	-0.959	0.370
	dSTR	0.738	0.484	-0.645	0.540
	NAcc	0.059	0.955	-0.727	0.491
	AMY	-1.17	0.279	-5.10	0.001*
	HPC	0.734	0.490	-1.28	0.241

*: Statistical significance

Table S4. A summary of two-way ANOVA in HPLC assays of monoamine metabolites.

		Strain		Density		Interaction	
		$F_{1,27}$	*p value*	$F_{1,27}$	*p value*	$F_{1,27}$	*p value*
**DOPAC/DA**	*PFC*	7.36	0.011*	2.13	0.156	1.40	0.246
	dSTR	8.39	0.007*	0.360	0.553	0.051	0.823
	NAcc	4.39	0.046*	0.591	0.449	0.173	0.680
	AMY	1.60	0.217	0.647	0.428	0.849	0.365
	HPC	2.62	0.117	0.758	0.392	1.18	0.288
**HVA/DA**	*PFC*	0.489	0.490	4.47	0.044*	0.394	0.536
	dSTR	4.60	0.041*	0.692	0.413	0.021	0.886
	NAcc	1.07	0.310	0.182	0.673	0.576	0.455
	AMY	0.079	0.780	0.295	0.591	1.20	0.282
	HPC	1.53	0.228	0.760	0.391	2.38	0.135
**5-HIAA/5-HT**	*PFC*	2.45	0.129	0.740	0.397	1.88	0.181
	dSTR	0.002	0.966	0.442	0.512	0.367	0.550
	NAcc	0.078	0.782	2.39	0.133	0.247	0.623
	AMY	1.91	0.178	2.23	0.147	0.835	0.369
	HPC	2.78	0.107	2.06	0.163	0.178	0.677

*: Statistical significance

Table S5. A summary of one sample t-test for the ratios of monoamine metabolites in mice at under 8mpc over 2mpc (8mpc/2mpc).

		C57		DBA	
		t_7	*p value*	t_7	*p value*
**DOPAC/DA**	*PFC*	-3.43	0.011*	-2.22	0.062
	dSTR	-0.697	0.508	-0.578	0.581
	NAcc	-1.71	0.130	-1.21	0.266
	AMY	-1.28	0.241	0.913	0.392
	HPC	-1.86	0.112	1.19	0.271
**HVA/DA**	*PFC*	-15.0	<0.001*	-6.41	<0.001*
	dSTR	-1.42	0.200	-1.23	0.260
	NAcc	-3.13	0.017*	0.315	0.762
	AMY	-2.23	0.061	0.905	0.396
	HPC	-6.23	<0.001*	1.42	0.200
**5-HIAA/5-HT**	*PFC*	-0.767	0.468	-1.31	0.232
	dSTR	-2.15	0.069	-0.055	0.957
	NAcc	-2.14	0.070	-4.23	0.004*
	AMY	-2.66	0.033*	-4.26	0.004*
	HPC	-1.77	0.128	-2.20	0.064

*: Statistical significance

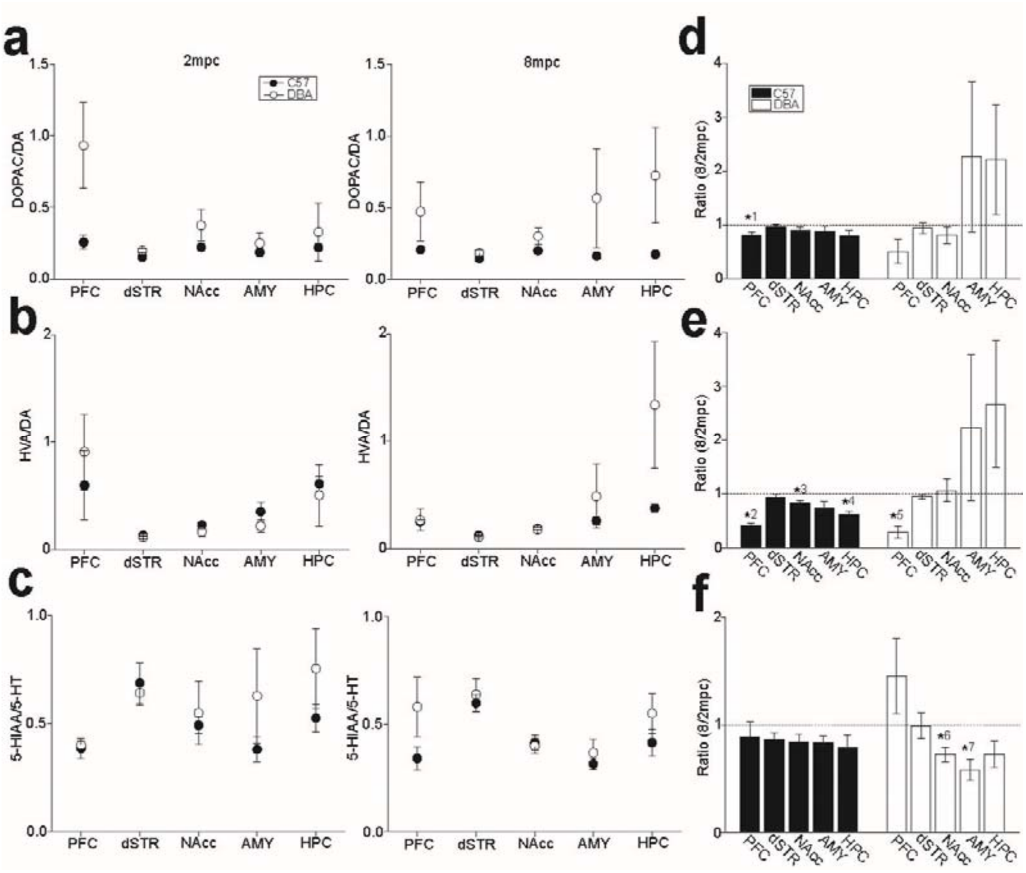

Supplementary Figure S1. Monoamine metabolites in corticolimbic regions of group-housed mice. **(a-c)** Graphs showing the ratios of DOPAC/DA (a) and HVA/ DA (b), and 5-HIAA/5-HT (c), in the PFC, dSTR, NAcc, AMY, and HPC of mice under 2mpc (left) and 8mpc (right). **(d-f)** Graphs showing the ratios of 8mpc over 2mpc for DOPAC/DA (d), HVA/DA (e), and 5-HIAA/5-HT (f). *^{1}p=0.011, *^{2}p<0.001, *^{3}p=0.017, *^{4}p<0.001, *^{5}p<0.001, *^{6}p=0.004, *^{7}p=0.004.

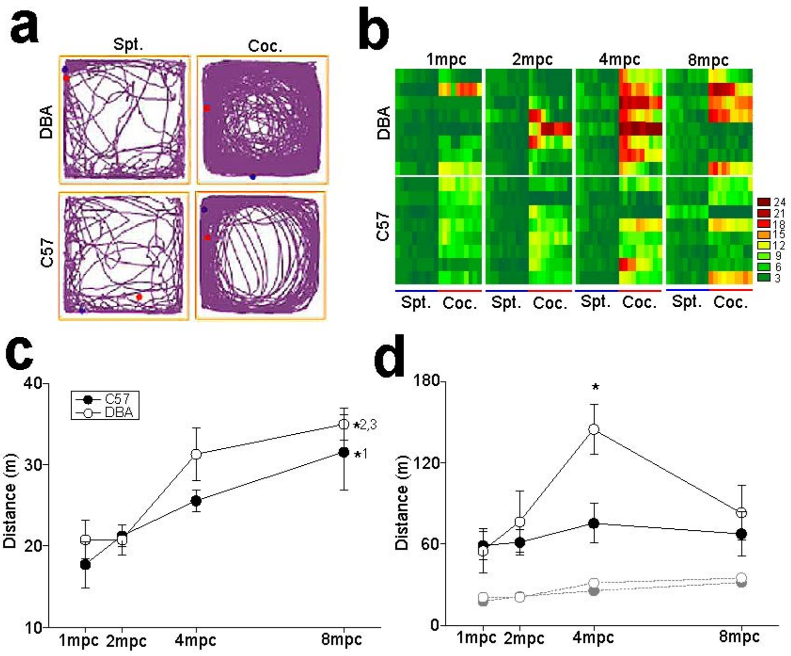

Supplementary Figure S2. The effects of housing density on novelty- and DA-dependent locomotion. **(a)** Examples of novelty- (spontaneous; spt.) and DA- (cocaine; coc.) dependent locomotion in C57 and DBA mice under 4mpc. **(b)** A diagram illustrating locomotor distance that each mouse travelled. Each mouse is aligned on y-axis, and x-axis indicates duration of recordings segmented into 1 minute each. **(c)** A graph showing spontaneous locomotor distance travelled. $*^1$p=0.012 for 1mpc vs. 8mpc in C57, $*^2$p=0.009 for 1mpc vs. 8mpc in DBA, $*^3$p=0.009 for 2mpc vs. 8mpc in DBA. **(d)** A graph showing locomotor distance travelled with cocaine administration. The dashed lines and circles with gray colors indicate spontaneous locomotion shown in (c). *p=0.007 for 1mpc vs. 4mpc in DBA.

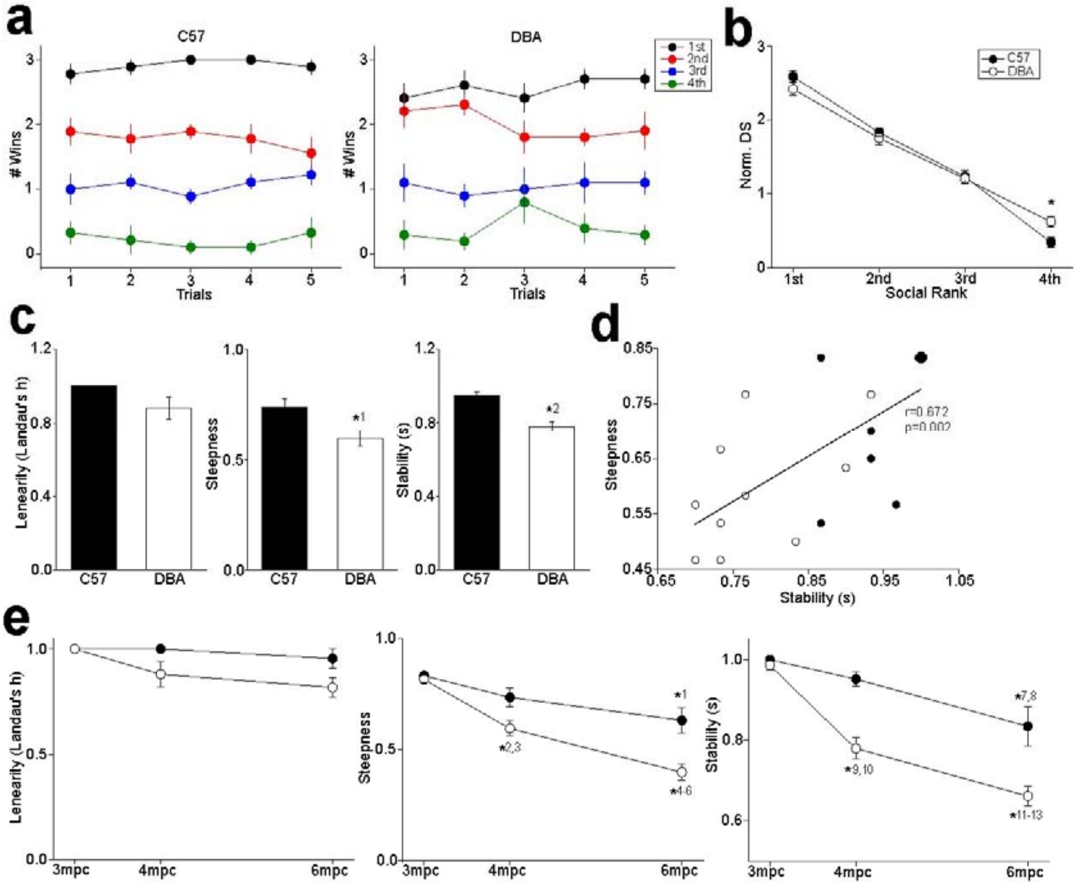

Supplementary Figure S3. Strain difference and the effects of housing density on social hierarchy. **(a)** Graphs showing the number of wins on each trial of tube test at 1st-4th rank C57 (left) and DBA (right) mice under 4mpc. **(b)** A graph showing normalized David's score (DS) calculated with the number wins/loss in tube test shown in (a). *p=0.009 for C57 vs. DBA under 4th rank. **(c)** Graphs showing Landau's corrected index (*h'*) for linearity (left), steepness (middle), and stability (*s*; right) of social hierarchy under 4mpc. *[1]p=0.022, *[2]p<0.001. **(d)** A graph showing a linear correlation between steepness and stability. **(e)** Graphs showing alterations of Landau's corrected index (*h'*) for linearity (left), steepness (middle), and stability (*s*; right) of social hierarchy under 3mpc, 4mpc, and 6mpc conditions. *[1]p=0.036 for 3mpc vs. 6mpc in C57, *[2]p=0.004 for 3mpc vs. 4mpc in DBA, *[3]p=0.050 for C57 vs. DBA under 4mpc, *[4]p<0.001 for 3mpc vs. 6mpc in DBA, *[5]p=0.013 for 4mpc vs. 6mpc in DBA, *[6]p=0.010 for C57 vs. DBA under 6mpc, *[7]p=0.005 for 3mpc vs. 6mpc in C57, *[8]p=0.038 for 4mpc vs. 6mpc in C57, *[9]p<0.001 for 3mpc vs. 4mpc in DBA, *[10]p<0.001 for C57 vs. DBA under 4mpc, *[11]p<0.001 for 3mpc vs. 6mpc in DBA, *[12]p=0.030 for 4mpc vs. 6mpc in DBA, *[13]p=0.003 for C57 vs. DBA under 6mpc.
